# *RCrane*: semi-automated RNA model building

**DOI:** 10.1107/S0907444912018549

**Published:** 2012-07-17

**Authors:** Kevin S. Keating, Anna Marie Pyle

**Affiliations:** a Department of Molecular, Cellular and Developmental Biology, Yale University, New Haven, CT 06511, USA; b Department of Chemistry, Yale University, New Haven, CT 06511, USA; c Howard Hughes Medical Institute, USA

**Keywords:** *RCrane*, RNA model building

## Abstract

RNA crystals typically diffract to much lower resolutions than protein crystals. This low-resolution diffraction results in unclear density maps, which cause considerable difficulties during the model-building process. These difficulties are exacerbated by the lack of computational tools for RNA modeling. Here, *RCrane*, a tool for the partially automated building of RNA into electron-density maps of low or intermediate resolution, is presented. This tool works within *Coot*, a common program for macromolecular model building. *RCrane* helps crystallographers to place phosphates and bases into electron density and then automatically predicts and builds the detailed all-atom structure of the traced nucleotides. *RCrane* then allows the crystallographer to review the newly built structure and select alternative backbone conformations where desired. This tool can also be used to automatically correct the backbone structure of previously built nucleotides. These automated corrections can fix incorrect sugar puckers, steric clashes and other structural problems.

## Abstract 2

*RCrane* is a new tool for the partially automated building of RNA crystallographic models into electron-density maps of low or intermediate resolution. This tool helps crystallographers to place phosphates and bases into electron density and then automatically predicts and builds the detailed all-atom structure of the traced nucleotides.

## 1. Introduction

In recent years, RNA crystal structures have contributed greatly to the understanding of numerous cellular processes (Ban *et al.*, 2000 ▶; Batey *et al.*, 2004 ▶; Selmer *et al.*, 2006 ▶). However, these structural studies are complicated by the fact that RNA crystals normally diffract to lower resolutions than protein crystals (Keating & Pyle, 2010 ▶). This low-resolution diffraction results in unclear electron-density maps, which frequently lead to errors in the structure-determination process. For protein crystallography, numerous tools exist for automated and partially automated model building (Cowtan, 2006 ▶; Langer *et al.*, 2008 ▶; Terwilliger, 2003 ▶); however, computational tools for RNA crystallography are only beginning to emerge. These tools aid in detecting (Chen *et al.*, 2010 ▶) and correcting (Wang *et al.*, 2008 ▶) errors in crystallo­graphic models, interpreting electron density (Gruene & Sheldrick, 2011 ▶) and classifying specific substructures (Sarver *et al.*, 2008 ▶; Wadley *et al.*, 2007 ▶), but few tools are available to aid in constructing the initial crystallographic model (Hattne & Lamzin, 2008 ▶).

Here, we present *RCrane* (**R**NA **c**onstructed using **r**ot**a**meric **n**ucl**e**otides), a tool for semi-automated model building of RNA into electron-density maps of low or intermediate resolution. *RCrane* first helps crystallographers to place phosphate and base atoms into electron density and then automatically predicts and builds the all-atom structure of the traced nucleotides. The prediction and building protocols are based on techniques that have previously been shown to produce highly accurate structures (Keating & Pyle, 2010 ▶), but the computer-assisted phosphate- and base-placement algorithms are newly developed and thoroughly tested below.

**Figure 1:**
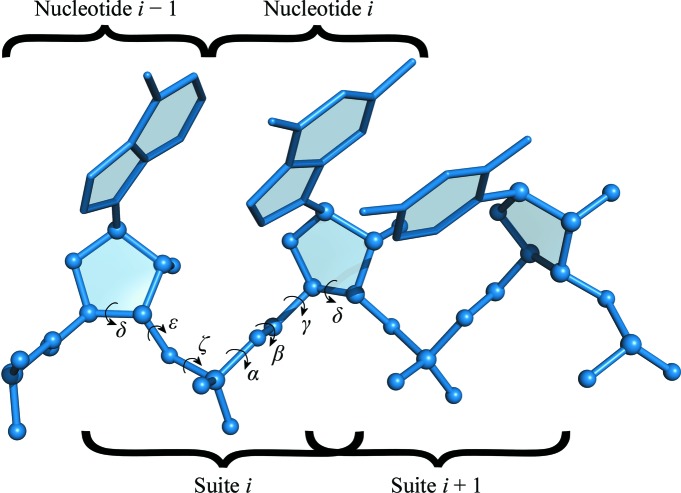
The RNA backbone, with suite and nucleotide divisions indicated. The backbone torsions belonging to suite *i* are also shown.

*RCrane* takes advantage of the recently developed consensus backbone conformer library (Richardson *et al.*, 2008 ▶). This conformer library enumerates roughly 50 discrete configurations for the RNA backbone and is comparable to the side-chain rotamer libraries frequently used in protein model building (Lovell *et al.*, 2000 ▶). It is important to note that the backbone conformer library divides the RNA backbone into suites rather than nucleotides, where each suite spans two sugars and the intervening phosphate (Fig. 1 ▶). The conformers are given two character names, such as 1a, where the first character represents the initial δ, ∊ and ζ torsions and the second character represents the α, β, γ and δ torsions. Additionally, any two adjacent suites overlap by one sugar (and the associated δ torsion), and therefore the ending pucker of one suite must be identical to the leading sugar pucker of the following suite.

*RCrane* also utilizes a modified pseudotorsional system (Keating & Pyle, 2010 ▶) that can classify RNA structure based solely on the phosphate and C1′ coordinates. These atoms can be accurately located in electron-density maps even when working at low or intermediate resolution. Phosphates are clearly visible owing to their high electron density. Conversely, the C1′ atom is difficult to locate directly; however, its location can be easily and uniquely determined from the coordinates of the nucleoside base (Keating & Pyle, 2010 ▶), which can be accurately located in density owing to its large size and rigidity. This minimal representation of RNA also holds potential for noncrystallographic applications such as molecular dynamics, where all-atom representations are not always possible owing to constraints in computing power (Cao & Chen, 2005 ▶; Jonikas *et al.*, 2009 ▶).

## 2. Program features

The functionality of *RCrane* can be divided into a number of major features. The initial model building consists of three main steps: backbone tracing, conformer prediction and coordinate calculation. Backbone tracing is carried out interactively using input from the crystallographer. The remaining two steps, however, are fully automated. After the initial model has been built, it may be reviewed and modified during alternate-conformer selection. *RCrane* also allows the crystallo­grapher to modify an existing structure. This feature can be used to correct structures built without *RCrane*, or it can be used to revise structures after crystallographic refinement. Additionally, *RCrane* works within *Coot*, a common program for macromolecular model building (Emsley *et al.*, 2010 ▶), and is designed to be intuitively usable by crystallo­graphers familiar with the *Coot* interface.

### 2.1. Backbone tracing

**Figure 2:**
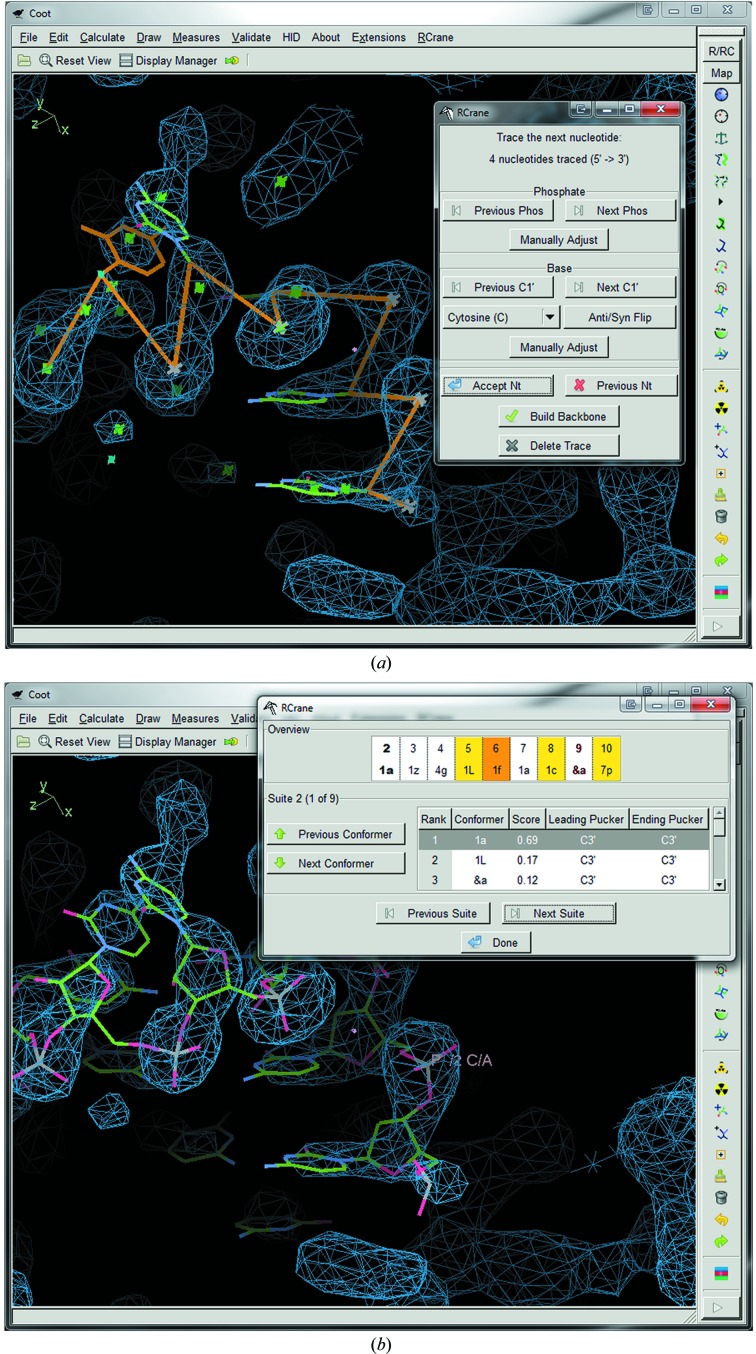
The *RCrane* interface. (*a*) *RCrane* assists the crystallographer in tracing phosphates and bases in the electron-density map. Here, the traced nucleotides are shown in green and orange, with the nucleotide that is currently being traced shown entirely in orange. The *RCrane* window on the right allows the user to select alternate phosphate and base locations. (*b*) After the backbone has been traced, *RCrane* automatically builds an all-atom model of the traced nucleotides. The user may then review the newly built structure and select alternate conformers where desired.

When constructing an initial model with *RCrane*, the first step is to build a backbone trace (Fig. 2 ▶ *a*), in which the phosphate and base atoms are placed into the electron density. In *RCrane* this step is performed interactively. The plugin provides suggestions for atom locations, but the crystallo­grapher must play an active role in the tracing process. The backbone trace begins with locating a single phosphate. When the user begins the trace, the plugin searches for all potential phosphate locations within 10 Å of the screen center (see §[Sec sec3]3). The phosphate closest to the screen center is initially selected, and the user may accept this phosphate location, select an alternate location, or manually adjust the phosphate coordinates.

Once the user accepts a phosphate location, *RCrane* will automatically trace a potential location for the next base and phosphate (see §[Sec sec3]3) and display these traced atoms. After the traced atoms are displayed, the user may accept the default coordinates, select an alternate base or phosphate location, or manually adjust the base or phosphate locations. The user must ensure that the traced atoms truly represent a base and phosphate, as density from metal ions can sometimes appear to be similar to density from phosphate groups. After the user accepts a set of coordinates, the next nucleotide is traced in a similar fashion. This process is repeated until the user has traced all of the desired connected nucleotides. Note that here ‘next’ may refer to either the 5′ or 3′ nucleotide depending on the directionality of the trace. This directionality is set by the user at the start of the trace.

### 2.2. Conformer prediction

After the backbone trace has been completed, *RCrane* must predict an appropriate conformer for each suite to be built. This step is performed automatically and does not require any user input. The prediction process is carried out as previously described (Keating & Pyle, 2010 ▶); however, six additional conformers are now considered during prediction (see §[Sec sec3]3). For each suite, this prediction assigns a score to each conformer. These scores sum to one for each suite and thus approximate a percentage likelihood for the given conformer. All scores are displayed during alternate-conformer selection (see below).

### 2.3. Coordinate calculation

**Figure 3:**
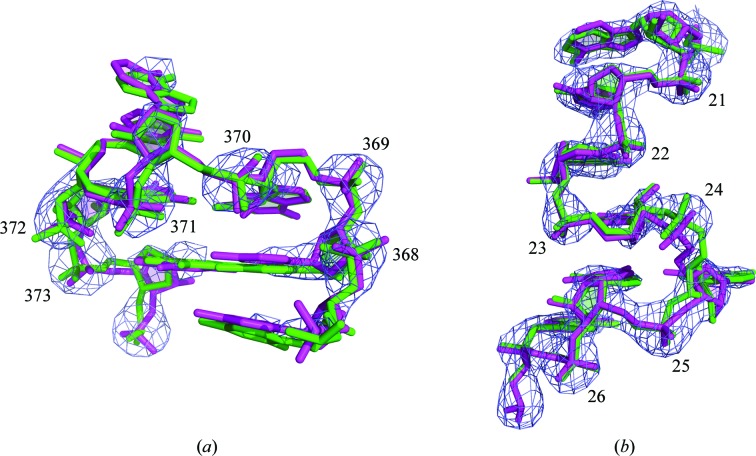
Coordinates built using *RCrane* are highly accurate. A comparison of models built using *RCrane* (magenta) and published coordinates (green) is shown. Suite numbers are as indicated. Note that the structures built using *RCrane* have not yet undergone crystallographic refinement. (*a*) The GANC tetraloop from the group II intron (Toor *et al.*, 2010 ▶). This structure was built into the 3.1 Å experimentally phased map (Toor *et al.*, 2008 ▶) shown contoured at 3.0σ. (*b*) An S-turn motif from the lysine riboswitch (Garst *et al.*, 2008 ▶) built into a 2.8 Å density map shown contoured at 1.8σ.

After conformer prediction, atomic coordinates must be calculated that match both the predicted conformer and the previously determined phosphate and base locations. As with conformer prediction, this step is performed automatically and does not require any user input. Coordinate calculation takes advantage of the minimization functions built into *Coot*. As a result, coordinate calculation is now approximately one order of magnitude faster than previously reported (Keating & Pyle, 2010 ▶). Additionally, the minimization procedure now incorporates information about the electron density. The resulting coordinates are still primarily determined using the predicted conformers and the traced phosphate and base locations; however, the inclusion of an electron-density term in the minimization helps to ensure that the calculated coordinates fit the density map as closely as possible (Fig. 3 ▶).

### 2.4. Alternate-conformer selection

After conformer prediction and coordinate calculation are complete, the user is presented with the newly built nucleotides and given the opportunity to review the structure (Fig. 2 ▶ *b*). The current conformers for each of the built suites are shown in the review window. This review window is colored using a ‘traffic-light’ color scheme, in which suites that should be reviewed by the crystallographer are highlighted in yellow, orange or red depending on the degree of uncertainty for that suite. This uncertainty may arise from either the conformer-prediction or the coordinate-calculation step. Uncertainty in conformer prediction occurs when the most likely and second most likely conformers are predicted to have similar likelihoods. Uncertainty in coordinate calculation occurs when the minimization procedure was unable to find an ideal match between the predicted conformer and the traced phosphate and base coordinates. Note that this ‘traffic-light’ color scheme is already used to present refinement results within *Coot* (Emsley *et al.*, 2010 ▶). Thus, *RCrane* and *Coot* present a consistent user interface for reviewing structural information.

This review window presents a list of all conformers and their conformer-prediction scores for the current suite (see above). This list is sorted by score and therefore presents likely alternate conformers at the top. From this window, the user can click on any alternate conformer and *RCrane* will automatically rebuild the affected nucleotides and display the results. If the alternate conformer requires a change in sugar pucker, adjacent suites will also be rebuilt to account for this pucker change. (Adjacent suites overlap by one sugar, so changing a sugar pucker in one suite implicitly changes a sugar pucker in an adjacent suite.) The review window thus allows the crystallographer to quickly examine a number of alternate conformers and select the most appropriate one.

### 2.5. Rotamerization

The previous features have been discussed in the context of building a new structure into electron density. However, rotamerization allows the user to correct or improve a region of an existing structure. This feature works similarly to the refine zone or regularize zone options in *Coot*, which carry out real-space refinement or geometry minimization on a specified region of a protein or nucleic acid structure. With rotamerization, the user first specifies a contiguous stretch of nucleotides. *RCrane* then uses the existing phosphate and base coordinates to carry out conformer prediction, coordinate calculation and alternate-conformer selection. This rebuilds the specified nucleotides and will typically correct errors in the backbone structure such as incorrect sugar puckers or steric clashes arising from incorrect backbone conformations.

This feature is useful when revising a model after a round of crystallographic refinement, as the refined map may provide more precise information about nucleotide structure. Rotamerization also provides a complement to services such as *MolProbity* (Chen *et al.*, 2010 ▶), which excels at detecting problem areas within crystallographic models but cannot offer specific fixes for RNA structures.

## 3. Methods

### 3.1. Phosphate picking

The initial step in an *RCrane* backbone trace is to compile a list of potential phosphate locations. This list is compiled using a peak-search function within *Coot*. This function is closely related to the *Coot* algorithm for water picking (*i.e.* finding electron density corresponding to water molecules). The phosphate search is carried out by first dividing the map into discrete regions of connected electron density. Each region corresponds to a single connected section of electron density and the size of these discrete regions is entirely dependent upon the map itself and the current contour level. At low contour levels these regions may be tens of ångströms long, while at higher contour levels these regions are typically only several ångströms in size. Within each region, the peak is defined as the point with the highest electron density. All peaks within the map are considered to be potential phosphate locations. This peak search is conducted with maps contoured at 1σ to 7σ in 0.25σ intervals and all duplicate peaks are ignored. This search is carried out only once per map and the results are cached for use in subsequent phosphate searches. When beginning a new backbone trace, all potential phosphate locations within 10 Å of the screen center are shown to the user and the initially selected phosphate is the peak closest to the screen center.

### 3.2. Nucleotide tracing

After the user has selected an initial phosphate, *RCrane* locates potential coordinates for the next base and phosphate. Here, ‘next’ and ‘previous’ are dependent on the direction of the backbone trace. When starting from phosphate *i* and tracing from 5′ to 3′, ‘next’ refers to base *i*, sugar *i* and phosphate *i* + 1. When tracing from 3′ to 5′, ‘next’ refers to base *i* − 1, sugar *i* − 1 and phosphate *i* − 1. Similarly, ‘previous’ refers to base *i* − 1, sugar *i* − 1 and phosphate *i* − 1 when tracing 5′ to 3′, and to base *i*, sugar *i* and phosphate *i* + 1 when tracing 3′ to 5′.

Phosphate candidates are located as described above and all phosphates within 10 Å of the starting phosphate (phosphate *i*) are considered to be potential next phosphates. For each potential next phosphate, a potential sugar center is found as follows. Let P_5′_ be the coordinates of the 5′ phosphate (*i.e.* the current phosphate when tracing 5′ to 3′ or the candidate phosphate when tracing 3′ to 5′) and let P_3′_ be the coordinates of the 3′ phosphate (*i.e.* the candidate phosphate when tracing 5′ to 3′ or the current phosphate when tracing 3′ to 5′). A cylindrical coordinate system is then defined with an origin at P_5′_ and a vertical (or cylindrical) axis of 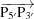. The polar (or azimuthal) axis is defined as an arbitrary vector orthogonal to 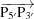 and starting at P_5′_.

**Figure 4:**
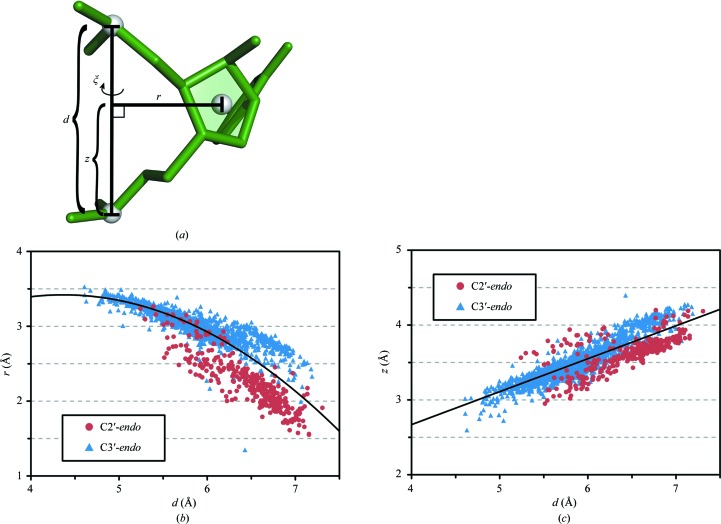
Two dimensions of the sugar-center location can be accurately predicted using only the coordinates of the 3′ and 5′ phosphates. (*a*) The interphosphate distance *d* is used to predict the radial and vertical components of the sugar-center location (*r* and *z*, respectively). (*b*) The radial component. Each point represents a nucleotide in the RNA05 data set, with sugar puckers as indicated. The quadratic regression (equation 1) is shown. For this regression, *r* ^2^ = 0.74. (*c*) The vertical component. The linear regression (equation 3) is shown. For this regression, *r* ^2^ = 0.78. Note that the regressions in (*b*) and (*c*) were calculated using all data points, regardless of sugar pucker.

Let G_candidate_ be the coordinates of the candidate sugar center in this cylindrical coordinate system, defined as (*r*, ξ, *z*) (Fig. 4 ▶ *a*). The radial component of the sugar-center location, *r*, is given by
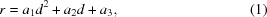
where *d* is the distance between the 5′ and 3′ phosphates,
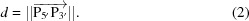
In (1), *a*_1_ = −0.186, *a*_2_ = 1.623 and *a*_3_ = − 0.124. These values were determined empirically using the RNA05 data set (Fig. 4 ▶ *b*). The vertical component of the sugar-center location, *z*, is given by
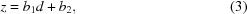
where *b*_1_ = 0.440 and *a*_2_ = 0.910. As above, these values were determined empirically using the RNA05 data set (Fig. 4 ▶ *c*). The azimuthal component of the sugar-center location, ξ, is defined such that it maximizes the electron density along the 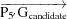 and 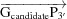 vectors.

**Figure 5:**
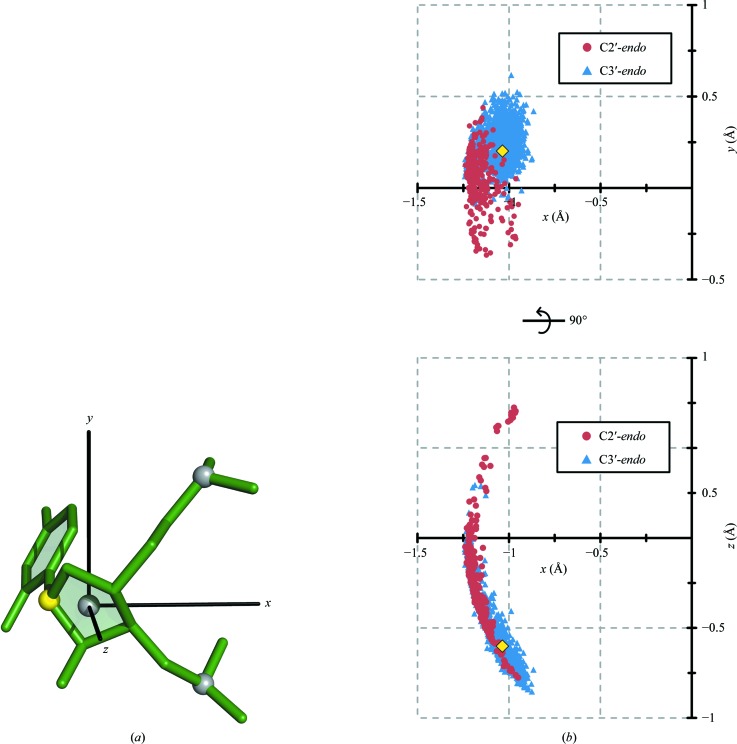
The coordinates of the C1′ atom can be accurately determined after locating the sugar center. (*a*) The Cartesian coordinate system defined using the locations of the sugar center, 3′ phosphate and 5′ phosphate (see §[Sec sec3]3). (*b*) Plots showing all C1′ atoms from the RNA05 data set relative to their respective sugar center, using the axes shown in (*a*) with sugar puckers as indicated. The top plot shows the C1′ coordinates in the *x* and *y* axes, while the bottom plot shows the *x* and *z* axes. The mean C1′ location is shown as a yellow diamond in both plots. During backbone tracing, *RCrane* places new C1′ atoms using this mean location.

These calculations define a single sugar center G_candidate_ for each phosphate candidate. G_candidate_ is then used to define C1′_candidate_, the candidate C1′ coordinate. To determine the location of C1′_candidate_, a Cartesian coordinate system is defined with the origin at G_candidate_ (Fig. 5 ▶ *a*). The *x* axis of this co­ordinate system is defined as the bisector of 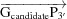 and 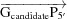 and the *y* axis is defined as 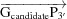 × 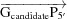. The *z* axis is then defined as the cross product of the *y* and *x* axes. The coordinates of C1′_candidate_ are then defined as (−1.036, 0.202, −0.601) in this coordinate system. These coordinates were determined empirically using the RNA05 data set (Fig. 5 ▶ *b*).

All phosphate candidates are then scored. When tracing the first nucleotide of a chain, the score for each candidate phosphate is given by
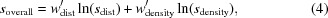
where *w*′_dist_ = 1, *w*′_density_ = 10 and the individual *s* terms are as defined below. Note that these candidate phosphates are the phosphates of nucleotide 2, as the phosphate of nucleotide 1 was placed during the initial phosphate picking. When tracing any subsequent nucleotide, the score for each candidate phosphate is given by 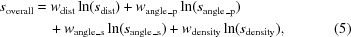
where *w*_dist_ = 5, *w*_angle_p_ = *w*_angle_s_ = 1, *w*_dist_ = 15 and the individual *s* terms are as defined below. Note that the values of all *w* and *w*′ terms were determined empirically.

The *s*_dist_ score is the likelihood of finding two successive phosphates at distance *d* in the RNA05 data set. The *s*_angle_p_ score is the likelihood of finding three successive phosphates at an angle ∠P_prev_P_current_P_candidate_ in the RNA05 data set, where P_prev_ are the coordinates of the previous phosphate, P_current_ are the coordinates of the current phosphate and P_candidate_ are the coordinates of the candidate phosphate. The *s*_angle_s_ score is the likelihood of finding successive C1′_*i*−1_, phosphate and C1′ atoms at an angle ∠C1′_prev_ *P*_current_C1′_candidate_ in the RNA05 data set, where C1′_prev_ are the coordinates of the previous C1′ atom. The likelihood distributions for *s*_dist_, *s*_angle_p_ and *s*_angle_s_ were calculated using kernel smoothing with a Gaussian kernel. The distributions themselves are provided as Supplementary Material^1^. The *s*_density_ score is the sum of the electron-density values at ten evenly spaced points along 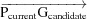, ten evenly spaced points along 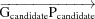, G_candidate_ and P_candidate_, with all electron-density values measured in e Å^−3^. Note that this *s*_overall_ scoring metric shares elements with the scoring proposed by Gruene & Sheldrick (2011 ▶).

**Figure 6:**
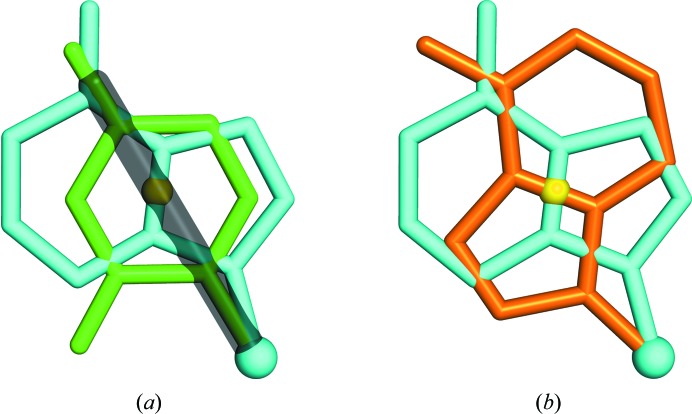
Positioning bases in density. (*a*) When placing bases into density, the nucleoside base is first approximated as a 5 Å vector, shown here in transparent gray. This vector is anchored at the C1′ candidate, which is shown as a cyan sphere. The vector is then replaced by a pyrimidine base, which may be computationally mutated to a purine if desired. To align the purine with the pyrimidine, the midpoint of the purine C4—C5 bond (yellow sphere) is aligned with the 5 Å vector. (*b*) When flipping a pyrimidine between *anti* and *syn* configurations, the base is rotated about the C1′ atom (cyan sphere) and the midpoint of the C4—C5 bond (yellow sphere). Flipping the base in this manner ensures that it is not moved out of the electron density.

The phosphate candidates are ranked based on their *s*_overall_ scores. The P_candidate_ and associated C1′_candidate_ with the highest score are used as the basis for the default next nucleotide. Before this nucleotide is displayed, however, base coordinates are calculated. A single 5 Å vector is used as a first approximation for the base, as this is roughly the distance between the C1′ and C4 atoms in pyrimidine (Fig. 6 ▶ *a*). The start of this vector is placed at C1′_candidate_. The endpoint of the vector, b_ℓ_, is then placed to maximize the density along 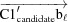 such that the angle between 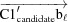 and the phosphate bisector is between 90° and 180°, inclusive.

A pyrimidine base is then placed along the vector such that the C1′ atom is located at C1′_candidate_ and the C4 atom is located along 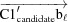. The base is then rotated about 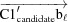 to maximize *s*_base_, where
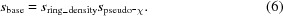
The *s*_ring_density_ score is the sum of the electron-density values at all ring atoms measured in e Å^−3^ (note that the O2 and N4/O4 atoms are ignored for this density fit). The *s*_pseudo-χ_ score serves to weight the base rotation towards typical *syn*, *anti* or high-*anti* values and is based on the pseudo-χ torsion angle, defined here for purines as the torsion of the phosphate, C1′, N9 and N1 atoms and for pyrimidines as the torsion of the phosphate, C1′, N1 and N3 atoms. The *s*_pseudo-χ_ score is defined as the likelihood of finding the current pseudo-χ value in the RNA05 data set, with near-zero likelihood values replaced by a floor value. This floor value prevents the exclusion of any strong fits to density owing to an unlikely pseudo-χ value. The likelihood distribution for *s*_pseudo-χ_ was calculated using kernel smoothing with a Gaussian kernel and the distribution is provided in the Supplementary Material^1^. After maximizing *s*_base_, the newly built base is computationally mutated to the user-specified base type. For purines, this mutation aligns the ring atoms. For pyrimidines, this mutation aligns 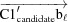 with the vector from C1′ to the midpoint of the C4—C5 bond while maintaining the plane of the base (Fig. 6 ▶ *a*). This ensures that the pyrimidine occupies roughly the same region of density as the purine.

If the crystallographer wishes to ‘flip’ a pyrimidine base between the *anti* and *syn* configurations, the base is rotated 180° about a vector from C1′ to the midpoint of the C4—C5 bond (Fig. 6 ▶ *b*). This ensures that the flipped base is not moved out of the electron density, as would occur if the base were rotated about χ. Purines, which are roughly symmetrical about the glycosidic bond, are simply rotated about χ.

### 3.3. Conformer prediction

The initial publication of the consensus conformer library (Richardson *et al.*, 2008 ▶) included eight ‘wannabe’ conformers that nearly satisfied the conformer-selection criteria. In the forthcoming update to the consensus conformer library, six of these ‘wannabe’ conformers have been promoted to full conformer status (Jain & Richardson, 2011 ▶). *RCrane* includes these six promoted conformers (2g, 2u, 2z, 3g, 5n and 5r) in its predictions. Other than the inclusion of these new conformers, conformer prediction is carried out as described previously (Keating & Pyle, 2010 ▶).

### 3.4. Coordinate calculation

The coordinate-calculation procedure used in *RCrane* is based on the previously reported minimization protocol (Keating & Pyle, 2010 ▶). However, there are a number of important differences in the new protocol. In* RCrane*, *Coot*’s built-in minimizer is used for coordinate calculation in place of a simplex minimizer. *Coot*’s minimizer uses the conjugate-gradient minimization algorithm implemented in the GNU Scientific Library (Emsley *et al.*, 2010 ▶; Galassi *et al.*, 2009 ▶). Additionally, the sugar ring is no longer treated as a rigid element. Instead, strong restraints are placed on the ν_0_, ν_1_ and ν_4_ torsions, with the ideal values of these restraints being dependent upon the sugar pucker. The phosphate atom is also no longer fixed during minimization. Instead, a strong harmonic restraint is used to keep the atom close to its starting position. The minimization also now includes a weak term measuring the match between the structure and the electron-density map. A weight of 10 is used for this term, which is significantly weaker than the default weight of 60 typically used in *Coot*.

### 3.5. Rotamerization

During rotamerization, conformer prediction is carried out as above. However, additional 5′ and 3′ heminucleotides are included during coordinate calculation. Torsion values from the existing structure are used as the torsion-restraint values for these heminucleotides. Such restraints are used for the α, β and γ torsions of the 5′ heminucleotide and the ∊ and ζ torsions of the 3′ heminucleotide. These restraints ensure proper geometry at the junction of the rotamerized nucleotides and the remainder of the structure.

### 3.6. Density maps

The results presented below make extensive use of two electron-density maps: the 3.1 Å resolution group II intron map (Toor *et al.*, 2008 ▶) and the 2.8 Å resolution lysine ribo­switch map (Garst *et al.*, 2008 ▶). In the group II intron map the quality of the experimental phases was quite high, which meant that nonhelical regions could be reliably built directly into the experimentally phased map. As such, the experimentally phased map was used here. While the lysine ribo­switch map was of higher resolution than the group II map, the experimental phases were of lower quality. As such, a model-phased map was calculated with *REFMAC*5 (Murshudov *et al.*, 2011 ▶) using the coordinates of 118 helical nucleotides from the published structure. This mimicked a map that would be generated from crystallo­graphic refinement after manually placing the helical regions of the structure.

## 4. Results and discussion

### 4.1. Backbone tracing

Building RNA structure using *RCrane* consists of three main tasks: backbone tracing, conformer prediction and coordinate calculation. Backbone tracing can be further divided into four steps: locating potential phosphates, locating sugar centers, locating C1′ atoms and locating bases. First, potential phosphates are located by searching the electron-density map for strong peaks of density that may correspond to phosphates (see §[Sec sec3]3). In a previous study (Keating & Pyle, 2010 ▶), we showed that accurate conformer predictions required phosphate coordinates within 1–1.5 Å of their published locations. To test the newly developed phosphate-search technique, we assessed how many phosphates within the group II intron (Toor *et al.*, 2010 ▶) and lysine riboswitch (Garst *et al.*, 2008 ▶) structures had nearby electron-density peaks. In the experimentally phased group II intron map, 290 of 388 phosphates (75%) had a density peak within 1.0 Å, 306 phosphates (79%) had a density peak within 1.5 Å and 82 phosphates (21%) had no peaks within 1.5 Å. Additionally, there were 524 density peaks that were further than 1.5 Å from a published phosphate location. These peaks primarily corresponded to the published locations of ribose sugars, nucleoside bases, coordinated metal ions and structured water molecules. While this presents a large number of false-positive peaks, subsequent backbone-tracing steps serve to filter out these false positives and help the user to select peaks that correspond to true phosphate locations. The results were similar when using the model-phased map (see §[Sec sec3]3) of the lysine riboswitch. In this map, 118 of 161 phosphates (73%) had a peak within 1.0 Å, no additional phosphates had a peak within 1.5 Å, 43 phosphates (27%) had no peak within 1.5 Å and 410 peaks were further than 1.5 Å from a published phosphate location.

When tracing the backbone, *RCrane* next calculates potential sugar center coordinates between pairs of potentially adjacent phosphates. To do this, the *r* and *z* coordinates of the sugar center are first predicted using the interphosphate distance (Fig. 4 ▶). These two values are calculated using regressions (see §[Sec sec3]3) determined from the RNA05 data set (Richardson *et al.*, 2008 ▶). Despite the simplicity of these calculations, the sugar center can be accurately located in the *r* and *z* dimensions. For nucleotides in RNA05, the average error in the *r* and *z* dimensions is 0.11 Å. These regressions are more accurate for C3′-*endo* nucleotides than for C2′-*endo* nucleotides. For C3′-*endo* nucleotides in RNA05 the average error is 0.09 Å, while for C2′-*endo* nucleotides the average error is 0.39 Å.

It should be noted that the relationship between *d* and *r* shown in Fig. 4 ▶(*b*) is best described by two separate linear regressions: one for the C3′-*endo* nucleotides and one for the C2′-*endo* nucleotides. However, during nucleotide tracing the sugar pucker is unknown. Therefore, these data were fitted using a single quadratic regression (see §[Sec sec3]3) that adequately describes all nucleotides. Additionally, it should be noted that the correlation between *d* and the sugar-center location is stronger than the correlation between *d* and the C1′ location, as the C1′ coordinates are more sensitive to the sugar pucker (data not shown). This indicates that the sugar center may prove to be a useful anchor point for other modeling applications.

Next, the appropriate value of ξ (Fig. 4 ▶ *a*) is determined using the electron density (see §[Sec sec3]3). This provides a single sugar-center location for each potential pair of adjacent phosphates. The C1′ coordinates can then be calculated from the sugar center using vector addition in a local Cartesian coordinate system (Fig. 5 ▶; see §[Sec sec3]3). As above, these calculations were determined using the RNA05 data set (Richardson *et al.*, 2008 ▶). For nucleotides in RNA05, the average error in estimating the C1′ location from the sugar center is 0.17 Å. Again, these calculations are more accurate for C3′-*endo* nucleotides than for C2′-*endo* nucleotides. For C3′-*endo* nucleotides the average error is 0.14 Å, while for C2′-*endo* nucleotides the average error is 0.46 Å.

As a further test of this C1′-finding method, we assessed how accurately C1′ atoms could be located in the group II intron and lysine riboswitch maps. First, all density peaks within 1.5 Å of a published phosphate location were located as described above. For all pairs of density peaks that corresponded to two adjacent phosphates, the coordinates of these peaks and the electron-density map were used to predict a potential C1′ location. This predicted location was then compared with the published C1′ coordinates. In the group II intron density map this resulted in 265 predicted C1′ locations, with 213 (80%) of these predictions within 1.0 Å of the published coordinates and 252 (95%) within 1.5 Å of the published coordinates. In the lysine riboswitch map there were 101 predicted C1′ locations, with 100 (99%) within 1.0 Å of the published coordinates and all predicted locations within 1.2 Å of the published coordinates.

After determining a C1′ location, *RCrane* next builds a base into the density adjacent to the C1′ atom (see §[Sec sec3]3). As shown previously (Keating & Pyle, 2010 ▶), conformer prediction is largely insensitive to imprecision in the base coordinates. However, accurate base positioning is clearly important for crystallographic refinement and interpretation of the resulting structure. The base-building method was tested using the group II intron and lysine riboswitch maps. For all C1′ co­ordinates that were predicted within 1.5 Å of their published location, a base position was predicted and built using the appropriate base type (*i.e.* A, C, G or U). The r.m.s.d. was then calculated between the predicted and published base coordinates. In the group II intron map 252 bases were built. Of these bases, 123 (49%) were within 1.0 Å r.m.s.d. of their published coordinates and 213 (85%) were within 2.0 Å r.m.s.d. In the lysine riboswitch map 101 bases were built, with 74 bases (74%) within 1.0 Å r.m.s.d. and all bases within 1.9 Å r.m.s.d.

### 4.2. Conformer prediction and coordinate calculation

After backbone tracing is complete, *RCrane* must predict an appropriate conformer for each suite and then calculate atomic coordinates that match both the predicted conformer and the traced phosphate and base locations. Conformer prediction and coordinate calculation were carried out as previously described (Keating & Pyle, 2010 ▶) with only minor modification (see §[Sec sec3]3). The additional conformers considered during conformer prediction are rare and thus have a negligible impact on conformer-prediction accuracy (data not shown). The modifications to the coordinate-calculation procedure serve to dramatically speed up the minimization process, but do not produce substantially different coordinates. Both conformer prediction and coordinate calculation have previously been shown be highly accurate (Keating & Pyle, 2010 ▶): one of the first three conformer predictions was correct 98% of the time and the first prediction was correct 84% of the time. Coordinate calculation built suites that matched the intended conformer 97.6% of the time, with many of the remaining 2.4% of suites containing only imperceptible differences to their target conformer (Keating & Pyle, 2010 ▶). These steps are not explicitly further tested here.

### 4.3. Building RNA using *RCrane*

Even when using *RCrane*, building an RNA structure into electron density is a complex process. To describe how *RCrane* fits into this procedure, we present here a general workflow for crystallographic RNA model building. It should be noted that this is a simplified and idealized description and that model building rarely proceeds entirely according to plan. The first step is to locate regions of the map that correspond to helical RNA. These regions can typically be identified even when the phase quality is poor. Additionally, RNA helical structure is highly regular, so these nucleotides can be accurately built even when the density is imprecise. This building can be performed using *RCrane*. Alternatively, if the experimental phases are so poor that locating individual phosphates and bases is difficult, then five to ten nucleotide stretches of idealized helical geometry can be manually docked into the density. Frequently, the crystallographer can then assign sequence to many of these newly built helices using the secondary structure, which is typically well characterized prior to crystallization.

After building the helices, a round of crystallographic refinement will dramatically improve the quality of the phases, resulting in a noticeably clearer density map. Next, nonhelical regions of the RNA can be built using *RCrane*. At this point, the length and sequence of many of these single-stranded regions is known. The building process then continues in an iterative manner, with improved phases helping to place new regions of structure and new regions of structure helping to improve phase quality.

### 4.4. Test cases

To further demonstrate the *RCrane* building process, we present a detailed description of building two nonhelical regions of RNA using *RCrane*: the GANC tetraloop of the group II intron (Keating *et al.*, 2008 ▶) and an S-turn motif in the lysine riboswitch (Garst *et al.*, 2008 ▶). This building was carried out without referring to the published coordinates. The GANC tetraloop (Fig. 3 ▶ *a*) was built starting at the phosphate of nucleotide 367 and the structure was traced 5′ to 3′. When building the initial nucleotide, the third-choice phosphate location was used (*i.e.* the ‘Next Phos’ button was clicked twice), as *RCrane* initially began to trace in the direction of the helix rather than the tetraloop. When building the first nucleotide, *RCrane* has no existing structure or directional information to inform its initial backbone placement; hence, the first-choice phosphate location may be incorrectly placed. After this initial nucleotide, however, the default locations were used for all bases and phosphates. In total, seven nucleotides were traced.

After conformer prediction and coordinate calculation were carried out automatically, suite 372 was flagged as uncertain (colored orange) during alternate-conformer selection. The most likely conformer for this suite was 1f, with a score of 0.40, while the second most likely conformer was 1a, with a score of 0.29. After examining the fit to density, this suite was changed to 1a. Additionally, 1a is the A-form helical conformer and therefore the most abundant backbone configuration. Conformer abundance is not considered during conformer prediction, but the abundance of 1a further supports its selection as an alternate conformer for this suite. No other suites were flagged as uncertain and so no other conformers were changed. The structure resulting from this building was highly accurate and closely matched the published GANC coordinates (Fig. 3 ▶ *a*).

Building of the S-turn motif was started at the phosphate of nucleotide 20 and the structure was traced 5′ to 3′. Despite the model phasing (see §[Sec sec3]3), this map was less precise than the group II intron map above, which led to erroneous default structure placement during backbone tracing. As a result, alternate phosphate locations were selected for most nucleotides (*via* the ‘Next Phos’ button). Additionally, nearly all base positions were manually adjusted by approximately 0.5 Å to better fit the density and to improve stacking on the already-built 5′ nucleotides. As above, seven nucleotides were traced in total. After fully automated conformer prediction and coordinate calculation, suite 22 was flagged as uncertain (colored orange) during alternate-conformer selection. For this suite, 1a was the most likely conformer and was the best fit to the density, so the conformer was not changed. As with the tetraloop above, the resulting structure closely matched the published coordinates (Fig. 3 ▶ *b*). Note that the base of nucleotide 24 was positioned incorrectly during backbone tracing by approximately 0.5 Å, leading to the slight discrepancy between the traced and published coordinates in this nucleotide.

From building into these and other maps, it is possible to make observations about the capabilities of *RCrane*. This building methodology appears to be more sensitive to phase quality than to map resolution. As a result, *RCrane* should be usable with X-ray crystallography density maps up to approximately 4 Å resolution provided that the phase quality is sufficient to generate an interpretable map. However, with X-ray crystallography calculating accurate experimental phases typically becomes more difficult as the resolution worsens. This points to the possibility of using *RCrane* with cryo-electron microscopy (cryo-EM) data, as phase information can be directly and accurately measured from cryo-EM experiments. Additionally, the resolutions achieved *via* these experiments are continuously and rapidly improving (Seidelt *et al.*, 2009 ▶; Yu *et al.*, 2008 ▶). It is therefore possible that the *RCrane* methodology could soon be used to build a *de novo* all-atom structure into a cryo-EM density map. Further work in this area is planned.

Although there are relatively few tools designed specifically for crystallographic RNA model building, recent releases of *ARP*/*wARP* are able to build nucleic acids into electron-density maps in an automated fashion (Hattne & Lamzin, 2008 ▶). This building technique has been tested with protein–nucleic acid complexes, where it has been shown to aid in model building (Hattne & Lamzin, 2008 ▶). In order to compare model building in *RCrane* with that in *ARP*/*wARP*, we used *ARP*/*wARP* to build into the electron-density maps of the group II intron and lysine riboswitch. For the group II intron, *ARP*/*wARP* was able to automatically build 191 of 388 (49%) nucleotides; however, the built nucleotides contained highly distorted geometry. Of the 191 built nucleotides, 182 (95%) contained a steric clash greater than 0.4 Å, 127 (66%) contained a steric clash greater than 1.0 Å and 17 (9%) contained a steric clash greater than 1.5 Å, as measured using *MolProbity* (Chen *et al.*, 2010 ▶). Additionally, many of the bases were built in incorrect locations, with only 53 of the 191 built bases (28%) within 2.0 Å r.m.s.d. of their published locations. Furthermore, 35 nucleotides (18%) were placed into density backwards (*i.e.* running 3′ to 5′ instead of 5′ to 3′). Owing to the severity of these issues, this automatically built structure would not provide a suitable starting point for further structure building or for crystallographic refinement.

For the lysine riboswitch map, *ARP*/*wARP* was able to build 120 of 161 nucleotides; however, the built structure again contained highly distorted geometry. Of the 120 built nucleotides, 91 (76%) contained a steric clash greater than 0.4 Å, 29 (24%) contained a steric clash greater than 1.0 Å and seven (6%) contained a steric clash greater than 1.5 Å (Chen *et al.*, 2010 ▶). Base placement was dramatically improved relative to the group II intron building, as 91 of the 120 bases (76%) were within 2.0 Å r.m.s.d. of their published coordinates. However, 18 lysine riboswitch nucleotides (15%) were placed into density backwards. Of the 120 built nucleotides, 106 corresponded to helical nucleotides that were used to calculate the map (see §[Sec sec3]3). The remaining 14 nucleotides consisted of eight segments, each of one to three nucleotides in length. All eight segments corresponded to nucleotides present in the published structure, indicating that they had been placed appropriately; however, the base planes of many of these nucleotides were approximately 45–90° away from their published orientations. Additionally, *ARP*/*wARP* failed to build the remaining 29 nonhelical nucleotides. Therefore, even when using a model-phased map, extensive interactive building would be necessary after the use of *ARP*/*wARP*.

These *ARP*/*wARP* results serve to highlight the difficulties in structure building for RNA crystallography. At first glance, the task of building RNA structure into an RNA-only density map and the task of building RNA structure into a protein–RNA complex density map seem highly similar. However, *ARP*/*wARP* struggles with the RNA-only maps tested here, even though it is perfectly capable of building into protein–RNA complex maps (Hattne & Lamzin, 2008 ▶). This may, at least in part, be a consequence of phase quality. Protein structure can be built into protein–RNA complex maps first and then used to calculate accurate model phases. These model phases dramatically improve the quality of the RNA regions of the map. Particularly when there are far more amino acids than nucleotides in the complex, the complete protein structure is likely to provide significantly more phasing power than placing helices into an RNA-only map. As such, when building into RNA-only density maps, approaches such as *RCrane* remain necessary.

## 5. Conclusions

RNA model building is a complex and involved process, as shown by the difficulties experienced with *ARP*/*wARP* when building into RNA-only density maps. However, *RCrane* allows a crystallographer to build RNA structure quickly and accurately by partially automating the model-building process, even when working with low-resolution or intermediate-resolution data. This program assists a crystallographer in locating bases and phosphates within electron density. From this basic backbone trace, *RCrane* can automatically construct a highly accurate all-atom model. *RCrane* works within *Coot*, thus providing an easy-to-use GUI interface that most crystallographers are already familiar with. *RCrane* is freely available from http://pylelab.org/software and runs on Windows, Linux and OS X. *RCrane* will also be included with all *Coot* installations starting with *Coot* 0.7 and will be accessible *via* the Extensions menu. This program is still under active development and continued improvement to the software is planned. A tutorial video is also available from http://pylelab.org/software. Additionally, tests of *RCrane* with cryo-EM data are planned. As this building technique appears to be more sensitive to poor phase quality than to low resolution, it is possible that the *RCrane* methodology could soon be used to build all-atom structure into cryo-EM density maps.

## Supplementary Material

Supplementary material file. DOI: 10.1107/S0907444912018549/rr5014sup1.zip

## Footnotes

1 Supplementary material has been deposited in the IUCr electronic archive (Reference: RR5014). Services for accessing this material are described at the back of the journal.
